# Cardiac arrhythmias associated with immune checkpoint inhibitors: A comprehensive disproportionality analysis of the FDA adverse event reporting system

**DOI:** 10.3389/fphar.2022.986357

**Published:** 2022-11-04

**Authors:** Feifei Wang, Qi Wei, Xinan Wu

**Affiliations:** ^1^ Department of Pharmacy, Hefei BOE Hospital, Hefei, China; ^2^ Department of Cardiology, Lu’an Hospital of Traditional Chinese Medicine, Lu’an, China

**Keywords:** immune checkpoint inhibitors, adverse event reporting system, cardiac arrhythmias, CTLA-4, PD-1, PD-L1

## Abstract

**Introduction:** With the widespread application of Immune checkpoint inhibitors (ICIs), it is important to explore the association between ICIs and cardiac arrhythmias and to characterize the clinical features of ICI-associated cardiac arrhythmias in real-world studies.

**Objective:** The purpose of this study was to characterize the main features of ICI-related cardiac arrhythmias.

**Methods:** From January 2017 to June 2021, data in the Food and Drug Administration Adverse Event Reporting System (FAERS) database were retrieved to conduct the disproportionality analysis. For the ICI-related cardiac arrhythmia detection, signals were detected by reporting odds ratio (ROR) and information component (IC), calculated using two-by-two contingency tables The clinical characteristics of patients reported with ICI-related cardiac arrhythmias were compared between fatal and non-fatal groups, and the time to onset (TTO) following different ICI regimens was further investigated. Multivariate logistic regression was used to evaluate the association between concurrent cardiotoxicities and ICI-associated arrhythmias.

**Results:** We identified a total of 1957 ICI–associated cardiac arrhythmias reports which appeared to influence more men (64.44%) than women (30.76%), with a median age of 68 [interquartile range (IQR) 60–75] years. Cardiac arrhythmias were reported most often in patients with lung, pleura, thymus and heart cancers (38.02% of 1957 patients). Compared with the full database, ICIs were detected with pharmacovigilance of cardiac arrhythmias (ROR025 = 1.16, IC025 = 0.19). Anti-PD-1 and anti-PD-L1 monotherapies were found to be related to higher reporting of arrhythmias, corresponding to ROR025 = 1.03, IC025 = 0.06 and ROR025 = 1.27, IC025 = 0.29, respectively, with the exception of anti-CTLA-4 monotherapies (ROR025 = 0.57, IC025 = −1.21). The spectrum of arrhythmias induced by ICIs differed among therapeutic regimens. There was no significant difference in the onset time between monotherapy and combination regimen. Moreover, reports of ICI-associated arrhythmias were associated with other concurrent cardiotoxicity, including cardiac failure [ROR 2.61 (2.20–3.09)], coronary artery disorders [ROR 2.28 (1.83–2.85)], myocardial disorders [ROR 5.25 (4.44–6.22)], pericardial disorders [ROR 2.76 (2.09–3.64)] and cardiac valve disorders [ROR 3.21 (1.34–7.68)].

**Conclusion:** ICI monotherapy and combination therapy can lead to cardiac arrhythmias that can result in serious outcomes and tend to occur early. Our findings underscore the importance of early recognition and management of ICI-related cardiac arrhythmias.

## Introduction

Immune checkpoint inhibitors (ICIs) are novel therapeutic agents that have revolutionized the treatment of numerous cancer types (Ferris et al., 2016; Reck et al., 2016; Larkin et al., 2019). ICIs target a range of costimulatory signaling molecules on T lymphocytes and antigen-presenting cells, such as cytotoxic T-lymphocyte antigen-4 (CTLA-4) and programmed cell death 1/ligand 1 (PD-1/PD-L1) (Mahmood et al., 2018; Ball et al., 2019).

Whereas, immune-related adverse events (irAEs) can affect multiple organ systems (Zhai et al., 2019; Hu et al., 2020; Mikami et al., 2021; Bomze et al., 2022), including the cardiovascular system (Salem et al., 2018; Ma et al., 2021). Due to its rarity, primary evidence regarding ICIs-associated cardiac arrhythmias is derived from case reports (Katsume et al., 2018; Bukamur et al., 2019; Prevel et al., 2020; Alhumaid et al., 2021; Savarapu et al., 2021) and clinical trials (Joseph et al., 2021), which have not systematically focused on ICI-induced arrhythmias. Cardiac arrhythmias associated with ICIs have been reported to occur in the setting of myocarditis (Katsume et al., 2018), which implies that ICI-related arrhythmias may be associated with concurrent cardiotoxicity. Besides, the overviewed relationship between arrhythmias and ICIs, the spectrum of potential signals, the factors related to fatality, as well as the clinical information of ICI-associated arrhythmias remain unknown.

In this pharmacovigilance study, we investigated the FDA’s Adverse Event Reporting System (FAERS) to identify the association between arrhythmias and different ICI regimens, detect a comprehensive spectrum of 17 potential signals, and present comprehensive information (patient characterizations, prognosis outcomes, the onset time and the association between concurrent cardiotoxicities and ICI-associated arrhythmias).

## Methods

### Data source

We conducted a retrospective pharmacovigilance study based on data from January 2017 to June 2021 in the FAERS database. The FAERS database is a spontaneous reporting system (SRS), which collects adverse events (AEs) reports by health professionals, consumers, pharmaceutical manufacturers, patients, and other non-healthcare workers. OpenVigil FDA, a pharmacovigilance tool, was adapted to extract FAERS data using the openFDA API for accessing the FDA drug-event database with the additional openFDA duplicate detection functionality.

### Procedures

The report of the FAERS database is coded using preferred terms (PTs) from Medical Dictionary for Regulatory Activities (MedDRA). We considered the following PTs as related to cardiac arrhythmias: “atrioventricular block complete (10003673),” “bundle branch block right (10006582),” “atrioventricular block (10003671),” “Bundle branch block left (10006580),” “arrhythmia (10003119),” “bradycardia (10006093),” “tachycardia (10043071),” “atrial fibrillation (10003658),” “sinus tachycardia (10040752),” “atrial flutter (10003662),” “sinus node dysfunction (10075889),” “supraventricular tachycardia (10042604),” “cardiac arrest (10007515),” “sudden death (10042434),” “ventricular tachycardia (10047302),” “cardio-respiratory arrest (10007617),” and “ventricular fibrillation (10047290).” The above PT level adverse events belonged to the following four High Level Terms (HLTs): “Cardiac conduction disorders (10000032),” “Rate and rhythm disorders NEC (10037908),” “Supraventricular arrhythmias (10042600),” and “Ventricular arrhythmias and cardiac arrest (10047283).” Concurrent cardiac AEs are entered using terms in the MedDRA terminology (provided in Supplementary Tables S1–S5). In this study, the following data concerning ICIs were retrieved from FAERS, including demographic information about the patient (e.g., gender, age), drug name, AEs and their outcomes, the country and year of reporting, the type of reporter, indications of use and the time to onset (TTO).

### Statistical analysis

We used descriptive statistics to present the clinical characteristics of the ICI-associated arrhythmias. A comparison of categorical variables was made between fatal and non-fatal group using the chi-squared test. We used *t* test and non-parametric test to analyze the normally distributed and not normally distributed continuous variables respectively, and *p* < 0.05 was considered significant. Multivariate logistic regression was used to examine concurrent cardiotoxicities related to ICI-related arrhythmias. The reporting odds ratio (ROR) with 95% confidence intervals (CIs) and Bayesian confidence propagation neural networks of information components (IC) were two specific indices to calculate disproportionality in pharmacovigilance (Noren et al., 2013; Zhai et al., 2019), which could detect potential signals in our investigation. The calculation formulas for ROR and IC are as follows:

$$ROR=\frac{N_{\text{observed}}+0.5}{N_{\text{expected}}+0.5}$$

$$95\%CI=e^{\text{In}\left(ROR\right)\pm 1.96\sqrt{\frac{1}{a}+\frac{1}{b}+\frac{1}{c}+\frac{1}{d}}}$$

$$N_{\text{expected}}=\frac{N_{\text{drug}}*N_{\text{event}}}{N_{\text{total}}}$$

$$IC=\log2\frac{N_{\text{observed}}+0.5}{N_{\text{expected}}+0.5}$$

$${IC}_{025}=IC-3.3\times {\left(N_{\text{observed}}+0.5\right)}^{-0.5}-2\times {\left(N_{\text{observed}}+0.5\right)}^{-1.5}$$

$${IC}_{975}=IC+2.4\times {\left(N_{\text{observed}}+0.5\right)}^{-0.5}-0.5\times {\left(N_{\text{observed}}+0.5\right)}^{-1.5}$$

*N*_expected_: the number of case reports expected for the target drug AEs. *N*_observed_: the observed number of case reports for the target drug AEs. *N*_drug_: the total number of case reports for the target drug, regardless of adverse reactions. *N*_event_: the total number of case reports for the target AEs, regardless of drug. *N*_total_: the total number of case reports in the database. For IC, a significant signal was considered when the lower limit of the IC 95% confidence interval (IC_025_) value was greater than zero (Bate et al., 1998; Noren et al., 2013). For ROR, a significant signal was considered when the lower end of the 95% credibility interval (ROR_025_) exceeded 1, with at least 3 cases (Rothman et al., 2004). One of the two algorithms meeting the criteria should be considered as a positive signal of arrhythmia. All the data analysis was performed by SPSS 24.0 (SPSS Inc, Chicago, IL, United States).

## Results

### Descriptive analysis

The FAERS database recorded 81,643 adverse events related to ICIs and 111,384 reports related to cardiac arrhythmias between 1 January 2017 and 30 June 2021. We identified 1957 reports of suspected ICI-related arrhythmias and summarized the clinical characteristics of patients in Table 1. The number of reported cases had gradually increased from 2017 to 2020. Males presented a larger proportion of arrhythmias than female patients (64.44% vs. 30.76%). The median age was 68 years [interquartile range (IQR) 60–75]. Physician submitted the highest number of case reports (808, 41.29%). The majority of reports were from North America (799, 40.83%), Europe (661, 33.78%) and Asian (388, 19.83%). Reports of ICI–associated arrhythmias were most frequently reported in lung, pleura, thymus and heart cancer patients (744, 38.02%). Nivolumab monotherapy generated the most reports associated with arrhythmias (659, 33.67%), followed by pembrolizumab monotherapy (479, 24.48%), and nivolumab plus ipilimumab (349, 17.83%). Only 77.77% of arrhythmias reports were isolated, with the overwhelmingly majority associated with concurrent cardiotoxicity, including cardiac failure (9.10%), coronary artery disorders (5.06%), myocardial disorders (9.66%), pericardial disorders (3.17%) and cardiac valve disorders (0.46%).

**TABLE 1:** Characteristics of patients with ICI-associated cardiac arrhythmias sourced from the FAERS database (January 2017 to June 2021).

Characteristics		Total reports, *n* (%)	Fatal cases, *n* (%)	Non-fatal cases, *n* (%)	*p*-value
Total		1957	617	1340	
Patient age (year)	—				*NS*
	Median (IQR)	68 (60-75)	69 (61-75)	68 (60-75)	
	< 18	28 (1.43%)	13 (2.11%)	15 (1.12%)	
	18–64	591 (30.20%)	174 (28.20%)	417 (31.12%)	
	65–74	631 (32.24%)	200 (32.41%)	431 (32.16%)	
	≥75	445 (22.74%)	146 (23.66%)	299 (22.31%)	
	Unknown	262 (13.39%)	84 (13.61%)	178 (13.28%)	
Gender	—				*0.009*
	Female	602 (30.76%)	160 (25.93%)	442 (32.99%)	
	Male	1261 (64.44%)	431 (69.85%)	830 (61.94%)	
	Unknown	94 (4.80%)	26 (4.21%)	68 (5.07%)	
Reporting year	—				*NS*
	2017	262 (13.39%)	88 (14.26%)	174 (12.99%)	
	2018	461 (23.56%)	151 (24.47%)	310 (23.13%)	
	2019	528 (26.98%)	160 (25.93%)	368 (27.46%)	
	2020	562 (28.72%)	174 (28.20%)	388 (28.96%)	
	2021	144 (7.36%)	44 (7.13%)	100 (7.46%)	
Tumor Indications	—				*0.002*
	Lung, pleura, thymus and heart	744 (38.02%)	262 (42.46%)	482 (35.97%)	
	Urinary system and male genital organs	310 (15.84%)	85 (13.78%)	225 (16.79%)	
	Digestive system	130 (6.64%)	59 (9.56%)	71 (5.30%)	
	Haematopoietic and lymphoid tissues	88 (4.50%)	28 (4.54%)	60 (4.48%)	
	Head and neck	61 (3.12%)	25 (4.05%)	36 (2.69%)	
	Endocrine organs	25 (1.28%)	3 (0.49%)	22 (1.64%)	
	Gynecologic organs	96 (4.91%)	19 (3.08%)	77 (5.75%)	
	Skin	267 (13.64%)	76 (12.32%)	191 (14.25%)	
	Central nervous system	15 (0.77%)	3 (0.49%)	12 (0.90%)	
	Unspecified or unknown indication	221 (11.29%)	57 (9.24%)	164 (12.24%)	
Area	—				*p < 0.001*
	Africa	4 (0.20%)	1 (0.16%)	3 (0.22%)	
	Asia	388 (19.83%)	163 (26.42%)	225 (16.79%)	
	Europe	661 (33.78%)	190 (30.79%)	471 (35.15%)	
	North America	799 (40.83%)	233 (37.76%)	566 (42.24%)	
	Oceania	47 (2.40%)	6 (0.97%)	41 (3.06%)	
	South America	54 (2.76%)	23 (3.73%)	31 (2.31%)	
	Unknown	4 (0.20%)	1 (0.16%)	3 (0.22%)	
Reporters	—				*NS*
	Physician	808 (41.29%)	273 (44.25%)	535 (39.93%)	
	Pharmacist	120 (6.13%)	32 (5.19%)	88 (6.57%)	
	Other health–professional	666 (34.03%)	194 (31.44%)	472 (35.22%)	
	Consumer or Non–health professional	344 (17.58%)	109 (17.67%)	235 (17.54%)	
	Unknown	19 (0.97%)	9 (1.46%)	10 (0.75%)	
ICI drug as suspected drug	—				*NS*
	Monotherapy	1579 (80.68%)	490 (79.42%)	1089 (81.27%)	*NS*
	Anti–CTLA–4 monotherapy	40 (2.04%)	12 (1.94%)	28 (2.09%)	
	Ipilimumab	37 (1.89%)	12 (1.94%)	25 (1.87%)	
	Tremelimumab	3 (0.15%)	0 (0.00%)	3 (0.22%)	
	Anti–PD–1 monotherapy	1145 (58.51%)	345 (55.92%)	800 (59.70%)	
	Nivolumab	659 (33.67%)	193 (31.28%)	466 (34.78%)	
	Pembrolizumab	479 (24.48%)	148 (23.99%)	331 (24.70%)	
	Cemiplimab	7 (0.36%)	4 (0.65%)	3 (0.22%)	
	Anti–PD–L1 monotherapy	394 (20.13%)	133 (21.56%)	261 (19.48%)	
	Atezolizumab	246 (12.57%)	81 (13.13%)	165 (12.31%)	
	Avelumab	51 (2.61%)	19 (3.08%)	32 (2.39%)	
	Durvalumab	97 (4.96%)	33 (5.35%)	64 (4.78%)	
	Combination therapy	378 (19.32%)	127 (20.58%)	251 (18.73%)	*NS*
	Ipilimumab+Nivolumab	349 (17.83%)	111 (17.99%)	238 (17.76%)	
	Tremelimumab+Durvalumab	29 (1.48%)	16 (2.59%)	13 (0.97%)	
Concurrent cardiotoxicity					*0.001*
	Cardiac failure	178 (9.10%)	59 (9.53%)	119 (8.85%)	
	Coronary artery disorders	99 (5.06%)	31 (5.01%)	68 (5.06%)	
	Myocardial disorders	189 (9.66%)	55 (8.89%)	134 (9.97%)	
	Pericardial disorders	62 (3.17%)	13 (2.10%)	49 (3.65%)	
	Cardiac valve disorders	9 (0.46%)	0 (0.00%)	9 (0.67%)	

Abbreviations: FAERS, Food and Drug Administration's Adverse Event Reporting System; ICI, immune checkpoint inhibitor; IQR: interquartile range; N: number of records.

As shown in Table 1, no significant differences were found in patient age, reporter and reporting year for fatal vs non-fatal reports. Use of different ICI regimens were similar in fatal vs. non-fatal ICI-related arrhythmia reports. There was a significant difference between fatal and non-fatal reports in tumor indications (*p* = 0.002), with the highest percentage of reported deaths (45.38%, 59/130) in digestive system patients. Notably, patient gender was statistically different between the two groups (*p* = 0.009), and the proportion of fatal reports in male patients was higher than that in female patients (69.85 vs. 25.93%). Concurrent cardiotoxicity was also different in fatal vs. non-fatal ICI-related arrhythmias reports (*p* = 0.001). Moreover, there was a significant difference in the reporting region between the two groups (*p* < 0.001), with the highest percentage of fatality occurring in South America (42.59%, 23/54).

### Signal values related to different immunotherapy regimens

In general, ICIs were significantly associated with the reporting frequency of arrhythmias [ROR 1.20 (1.16–1.24), IC025 0.19] (Table 2). Concerning reports of cardiac arrhythmias, a significant increased ROR was found for anti-PD-1 monotherapy [ROR 1.11 (1.03–1.21), IC025 0.06] and anti-PD-L1 monotherapy [ROR 1.37 (1.27–1.49), IC025 0.29], with the exception of anti-CTLA-4 monotherapy [ROR 0.62 (0.57–0.67), IC025 −1.21]. As for ICI combination therapy, nivolumab plus ipilimumab detected with pharmacovigilance signals of cardiac arrhythmias [ROR 1.52 (1.37–1.69), IC025 0.43], which was not seen in tremelimumab plus durvalumab [ROR 1.19 (0.82–1.72), IC025 −0.37]. Further analysis showed that combination regimen was associated with a higher risk of reports of cardiac arrhythmias compared with monotherapy [ROR 1.30 (1.20–1.41), IC025 0.21].

**TABLE 2:** Associations of different ICI regimens with cardiac arrhythmias.

	Drug	N	ROR	ROR_025_	ROR_975_	IC	IC_025_
Total	Total ICIs	1957	1.20	1.16	1.24	0.26	0.19
Monotherap	Anti–CTLA–4 monotherapy	40	0.62	0.57	0.67	−0.69	−1.21
	Ipilimumab	37	0.58	0.42	0.80	−0.81	−1.36
	Tremelimumab	3	3.57	1.07	11.97	1.84	−0.23
	Anti–PD–1 monotherapy	1145	1.11	1.03	1.21	0.16	0.06
	Nivolumab	659	1.13	1.04	1.22	0.17	0.04
	Pembrolizumab	479	1.11	1.01	1.21	0.15	0.00
	Cemiplimab	7	0.65	0.31	1.37	−0.62	−1.92
	Anti–PD–L1 monotherapy	394	1.37	1.27	1.49	0.46	0.29
	Atezolizumab	246	1.35	1.19	1.53	0.43	0.22
	Avelumab	51	1.68	1.27	2.22	0.75	0.28
	Durvalumab	97	1.30	1.06	1.59	0.38	0.04
Combination therapy	Combination therapy	378	1.49	1.37	1.62	0.57	0.40
	Ipilimumab+Nivolumab	349	1.52	1.37	1.69	0.60	0.43
	Tremelimumab+Durvalumab	29	1.19	0.82	1.72	0.25	−0.37
Anti-PD-1vs anti-ctla-4	—	1145	1.80	1.44	2.26	0.85	0.75
Anti-PD-L1vs anti-ctla-4	—	394	2.22	1.76	2.80	1.15	0.98
Anti-PD-1 vs anti-PD-L1	—	1145	0.81	0.75	0.88	−0.30	−0.40
Combination vs monotherapy therapy	—	378	1.30	1.20	1.41	0.38	0.21

Abbreviations N: number of records; ROR025: the lower end of the 95% confidence interval of ROR. ROR975: the upper end of the 95% confidence interval of IC; IC025: the lower end of the 95% confidence interval of IC.

### The signal spectrum of cardiac arrhythmias differs in immune therapies

The cardiac arrhythmia signal spectrum of different ICI strategies was shown in Table 3, where the IC 025 was regarded as an indicator. As shown in Table 3, ipilimumab plus nivolumab presented a broadest spectrum of cardiac arrhythmias AEs with 8 PTs detected as signals, ranging from cardiac arrest (IC 025 = 0.01) to sudden death (IC 025 = 1.90). For nivolumab, a total of 7 PTs as signals were observed, with signal values ranging from IC 025 = 0.19 (atrial fibrillation) to IC 025 = 1.34 (sudden death). There were 6 PTs both statistically associated with pembrolizumab and atezolizumab receiving. However, the drug with the least PTs were ipilimumab and cemiplimab, with no signal detected, followed by tremelimumab, with only one signal (tachycardia, IC 025 = 0.48) detected. Interestingly, both drugs (ipilimumab and tremelimumab) were all anti-CTLA-4 drugs, with no or only one reported AEs. Cemiplimab, as one of the three anti-PD-1 drugs, presented no signal due to the rare application.

**TABLE 3:** Arrhythmia Signal Profiles of Different ICI Strategies.

IC_025_>0	Ipilimumab	Tremelimumab	Nivolumab	Pembrolizumab	Cemiplimab	Atezolizumab	Avelumab	Durvalumab	Ipilimumab + Nivolumab	Tremelimumab + Durvalumab
Atrioventricular block complete	—	—	1.17	2.00	—	0.73	—	0.29	1.09	—
Bundle branch block right	—	—	−1.57	−1.67	—	—	—	—	−0.02	—
Atrioventricular block	—	—	0.50	0.18	—	−1.49	—	—	0.90	—
Bundle branch block left	—	—	—	−1.16	—	−0.63	—	—	−0.89	—
Arrhythmia	—	—	−0.33	−0.75	−1.03	−2.47	—	−2.70	−1.58	—
Bradycardia	—	—	−1.46	−1.70	—	−1.95	0.51	−2.96	−3.06	—
Tachycardia	−2.18	0.48	−0.89	−0.45	—	−0.83	−1.15	−0.58	−0.61	—
Atrial fibrillation	−0.66	—	0.19	0.12	—	0.80	−0.51	0.36	0.78	−2.80
Sinus tachycardia	—	—	−0.26	−0.61	—	0.14	—	−1.66	1.76	—
Atrial flutter	—	—	0.62	−0.44	—	0.53	—	—	0.24	1.35
Sinus node dysfunction	—	—	0.71	−1.84	—	−0.90	—	—	—	—
Supraventricular tachycardia	—	—	0.43	0.43	—	1.36	—	−1.17	0.42	—
Cardiac arrest	−1.38	—	−0.35	−1.03	—	−0.95	0.20	−0.49	0.01	0.47
Sudden death	—	—	1.34	1.34	—	1.08	1.17	−1.21	1.90	0.45
Ventricular tachycardia	—	—	−0.90	0.59	—	—	—	—	−0.61	—
Cardio-respiratory arrest	—	—	−0.54	−0.87	—	−1.00	—	−0.74	−1.59	—
Ventricular fibrillation	—	—	−1.32	−0.90	—	—	—	—	—	—

Atrioventricular block complete, atrial fibrillation and sudden death were three overlapping PTs. Among these, sudden death was the most frequent PT, also detected as the second strongest signal (IC 025 = 1.90). Both atrioventricular block complete and atrial fibrillation were found significantly associated with nivolumab, pembrolizumab, atezolizumab, avelumab, and ipilimumab plus nivolumab, with atrioventricular block complete detected as the strongest signal in pembrolizumab (IC 025 = 2.00).

### Time to cardiac arrhythmias onset

A total of 1353 ICI-associated cardiac arrhythmias reported TTO, as shown in Table 4 (There were few data on tremelimumab, which was not shown in Table 4). Among ICI monoregimens, we found no significant difference in the reporting onset time of cardiac arrhythmias (*p =* 0.061). The median time to onset was 47 days for ipilimumab (IQR 19–67), 35 days for nivolumab (IQR 12–135), 25 days for pembrolizumab (IQR 7–76), 56 days for cemiplimab (IQR 22–99), 34 days for atezolizumab (IQR 12–168), 18 days for avelumab (IQR 1–103), and 35 days for durvalumab (IQR 9–100), respectively. In addition, there was no significant difference in the onset time between monotherapy and combination regimen (ipilimumab vs. ipilimumab plus nivolumab, *p* = 0.606; nivolumab vs. nivolumab plus ipilimumab, *p* = 0.550; tremelimumab vs. tremelimumab plus durvalumab, *p* = 0.620; durvalumab vs. tremelimumab plus durvalumab, *p* = 0.061).

**TABLE 4:** Onset time of ICIs–associated arrhythmias.

	Ipilimumab (*n* = 37)	Tremelimumab ( *n*= 3)	Nivolumab (*n* = 659)	Pembrolizumab (*n* = 479)	Cemiplimab (*n* = 7)	Atezolizumab (*n* = 246)	Avelumab (*n*=51)	Durvalumab (*n* = 97)	Ipilimumab +Nivolumab (*n* = 349)	Tremelimumab +Durvalumab (*n* = 29)
Median (IQR)	47 (19–67)	143 (140–143)	35 (12–135)	25 (7–76)	56 (22–99)	34 (12–168)	18 (1–103)	35 (9-100)	31 (12-84)	135 (10–323)
0–30	8 (21.62%)	0 (0.00%)	214 (32.47%)	148 (30.90%)	2 (28.57%)	90 (36.59%)	25 (49.02%)	36 (37.11%)	128 (36.68%)	6 (20.69%)
31–60	9 (24.32%)	0 (0.00%)	79 (11.99%)	42 (8.77%)	0 (0.00%)	25 (10.16%)	7 (13.73%)	13 (13.40%)	49 (14.05%)	3 (10.34%)
61–90	4 (10.81%)	0 (0.00%)	26 (3.95%)	17 (3.55%)	1 (14.29%)	14 (5.69%)	0 (0.00%)	8 (8.25%)	23 (6.59%)	0(0.00%)
91–120	2 (5.41%)	0 (0.00%)	14 (2.12%)	8 (1.67%)	1 (14.29%)	8 (3.25%)	3 (5.88%)	3 (3.09%)	16 (4.58%)	1 (3.45%)
121–150	0 (0.00%)	1 (33.33%)	24 (3.64%)	6 (1.25%)	0 (0.00%)	6 (2.44%)	1 (1.96%)	3 (3.09%)	4 (1.15%)	3 (10.34%)
151–180	0 (0.00%)	2 (66.67%)	9 (1.37%)	8 (1.67%)	0 (0.00%)	3 (1.22%)	1 (1.96%)	1 (1.03%)	5 (1.43%)	1 (3.45%)
181–360	1 (2.70%)	0 (0.00%)	51 (7.74%)	19 (3.97%)	0 (0.00%)	23 (9.35%)	3 (5.88%)	8 (8.25%)	22 (6.30%)	3 (10.34%)
Greater than 360	1 (2.70%)	0 (0.00%)	42 (6.37%)	19 (3.97%)	0 (0.00%)	23 (9.35%)	4 (7.84%)	5 (5.15%)	14 (4.01%)	4 (13.79%)
Unknown	12 (32.43%)	0 (0.00%)	200 (30.35%)	212 (44.26%)	3 (42.86%)	54 (21.95%)	7 (13.73%)	20 (20.62%)	88 (25.21%)	8 (27.59%)

Abbreviations: N: number of records; IQR: interquartile range; ICI, immune checkpoint inhibitor.

### Associations between concurrent cardiotoxicity and ICI-associated arrhythmias

Table 5 showed the associations between concurrent cardiotoxicity reports and ICI-associated arrhythmias reports. In the multivariate logistic regression model, the following concurrent cardiotoxicities reports were associated with ICI-associated arrhythmias reports: cardiac failure (OR = 2.61, 95% CI 2.20–3.09, *p* < 0.001), coronary artery disorders (OR = 2.28, 95% CI 1.83–2.85, *p* < 0.001), myocardial disorders (OR = 5.25, 95% CI 4.44–6.22, *p* < 0.001), pericardial disorders (OR = 2.76, 95% CI 2.09–3.64, *p* < 0.001) and cardiac valve disorders (OR = 3.21, 95% CI 1.34–7.68, *p* < 0.001). As the MedDRA classification “cardiac arrhythmias” encompasses a broad range of diseases, we subgrouped the 1957 reports by the four MedDRA HLT classifications: “rate and rhythm disorders NEC,” “cardiac conduction disorders,” “ventricular arrhythmias,” and “supraventricular arrhythmias” for further analysis (the MedDRA abbreviation “NEC” denotes “Not Elsewhere Classified”). The associations between concurrent cardiotoxicity reports and specific arrhythmias reports under each HLT was diverse, with myocardial disorders having significantly elevated reporting of four specific arrhythmias in HLT level but cardiac valve disorders only increasing the risk of supraventricular arrhythmias.

**TABLE 5:** The multivariate analysis of concurrent cardiotoxicity and ICI-associated arrhythmias.

Concurrent cardiotoxicity	Arrhythmias		Cardiac conduction disorders		Rate and rhythm disorders NEC		Supraventricular arrhythmias		Ventricular arrhythmias and cardiac arrest	
	OR (95%CI)	*p-value*	OR (95%CI)	*p-value*	OR (95%CI)	*p-value*	OR (95%CI)	*p-value*e	OR (95%CI)	*p-value*
Cardiac failure	2.61 (2.20–3.09)	*p*<0.001	1.24 (0.73-2.12)	0.427	2.79 (2.02-3.84)	*p*<0.001	3.40 (2.68–4.32)	*p*<0.001	2.42 (1.79–3.28)	*p*<0.001
Coronary artery disorders	2.28 (1.83–2.85)	*p*<0.001	3.04 (1.81-5.11)	*p*<0.001	3.34 (2.32-4.80)	*p*<0.001	2.04 (1.45–2.89)	*p*<0.001	1.50 (0.96–2.34)	0.075
Myocardial disorders	5.25 (4.44–6.22)	*p*<0.001	35.51 (25.82-48.85)	*p*<0.001	2.45 (1.64-3.64)	*p*<0.001	2.73 (2.01–3.69)	*p*<0.001	5.90 (4.47–7.78)	*p*<0.001
Pericardial disorders	2.76 (2.09–3.64)	*p*<0.001	0.78 (0.24-2.54)	0.683	2.53 (1.49-4.31)	0.001	4.47 (3.18–6.28)	*p*<0.001	1.23 (0.65–2.35)	0.523
Cardiac valve disorders	3.21 (1.34–7.68)	*p*<0.001	—	—	0.80 (0.10-6.37)	0.833	5.79 (2.29–14.68)	*p*<0.001	1.82 (0.40–8.31)	0.441

Abbreviations: CI, confidence interval; OR, odds ratio; eGFR, estimated glomerular filtration rate.

## Discussion

To the best of our knowledge, this is the first pharmacovigilance study on cardiac arrhythmias reports associated with ICIs based on the FAERS database. Our research presented a comprehensive description on cardiac arrhythmias associated to different ICI regimens, resulting in certain systematical and accurate conclusions.

Importantly, our study detected a significant signal between cardiac arrhythmias and ICI therapy. Notably, our study revealed that immune-mediated arrhythmias were disproportionately more frequent reported in concurrent cardiotoxicity, which was concordant to what is observed in prior studies (Johnson et al., 2016; Salem et al., 2018; Herrmann, 2020; Nso et al., 2020; Baik et al., 2021; Stein-Merlob et al., 2021). The ICI-associated arrhythmias reports indicated a complicated clinical course, which prompted an evaluation for the presence of other cardiotoxicities. Similarly, all patients presenting with symptoms concerning for ICI-associated cardiotoxicity should have a 12-lead ECG to assess for arrhythmias.

In our study, concurrent cardiotoxicity increasing the reporting risk of ICI-related arrhythmias included cardiac failure [ROR 2.61 (2.20–3.09)], coronary artery disorders [ROR 2.28 (1.83–2.85)], myocardial disorders [ROR 5.25 (4.44–6.22)], pericardial disorders [ROR 2.76 (2.09–3.64)] and cardiac valve disorders [ROR 3.21 (1.34–7.68)]. Different types of reports of ICI-associated arrhythmia, including conduction delays, rate and rhythm disorders, supraventricular and ventricular arrhythmias (Escudier et al., 2017; Mir et al., 2018; Stein-Merlob et al., 2021), presented consistent association with concurrent myocardial disorders and different correlation with other four kinds of concurrent cardiotoxicity. Previous studies suggested that reports of supraventricular arrhythmias following ICI therapy were associated with other concurrent irAEs (Salem et al., 2018) or T-lymphocyte-mediated inflammation in the sinoatrial and atrioventricular nodes (Johnson et al., 2016; Nso et al., 2020). Rate and rhythm disorders reports (including arrhythmia, bradycardia and tachycardia) could be seen in the setting of high degrees of conduction block. In patients receiving ICI therapy, ventricular arrhythmias and conduction block might be a result of the T-lymphocyte-mediated inflammatory infiltration into the myocardium (Johnson et al., 2016; Herrmann, 2020; Baik et al., 2021; Stein-Merlob et al., 2021). Whereas, precise mechanisms underlying of ICI-associated arrhythmias remain to be elucidated. It is unclear whether the increased reporting of arrhythmias following ICI therapy was due to concurrent cardiotoxicities *versus* due to ICI treatment itself.

This study showed that ICI-associated arrhythmias were over-reported for anti-PD-1/PD-L1 vs anti-CTLA-4 monotherapy [ROR: 1.80 (1.44–2.26) and 2.22 (1.76–2.80), respectively]. In addition, there was increased risk of reports of arrhythmias with dual ICI combination therapy [ROR: 1.30 (1.20–1.41)]. Anti-CTLA-4 agents were not associated with over-reporting frequencies of reporting arrhythmias [ROR: 0.62 (0.57–0.67)], consistent with previous findings showing that anti-CTLA-4 was not associated with risk of reporting pericardial toxicities and less susceptible to myocarditis (Zhou et al., 2019; Ma et al., 2021). The non-susceptibility of anti-CTLA-4 to pericarditis and myocarditis was due to the difference of disease-specific effects (Salem et al., 2018; Ma et al., 2021) and mechanism (Grabie et al., 2019), respectively. Due to the correlation between ICI related arrhythmias and the both two concurrent cardiotoxicities, the low reporting risk of anti-CTLA-4 in myocarditis and pericarditis may lead to low reporting risk of arrhythmias. Owing to a lack of studies on immunotherapy-induced arrhythmias, the rationale for no signal for anti-CTLA-4 drugs need to be further elucidated and explored.

Our study showed that most reports of ICI-associated arrhythmias occurred early after ICI initiation, with no significant difference between different ICI regimens. The median time to onset of arrhythmias reports associated with ICIs was 32 (IQR 10–109) days, and most reports (33.57%) appeared within the first 30 days after the initiation of ICI, which suggested the importance of cardiac monitoring during the higher-risk time window of 30 days. The median onset time reported with dual ICI therapies were 32.5 (IQR 12–96.25) days, and no earlier onset of arrhythmias was reported with ICI combination therapies than with ICI monotherapies. This finding was inconsistent with those of previously published case reports of ICI-associated cardiotoxicity, which reported that cardiotoxicity occurred earlier when two ICIs were combined (Zhou et al., 2019).

This study involves certain limitations that should be recognized. Firstly, resulting from the signal mining of FAERS database, our study may be associated with inevitable underreporting and selective reporting. Firstly, as a spontaneous reporting system (SRS), there are some limitations inherent to FAERS database, including missing data, partial clinical features of AEs, and reporting bias (e.g., inevitable underreporting, selective reporting and the potential for the data to be misunderstood). Secondly, it is difficult to control for confounding factors such as history of arrhythmias or concomitant medications, both of which might influence the risk of cardiac arrhythmias. Lastly, due to lack of the number of patients exposed to ICIs without AEs, FAERS data can neither be used to calculate the incidence of an adverse reaction nor quantify adverse reaction signals based on the total number of AEs.

## Conclusion

This study comprehensively evaluated the relationship between ICIs and cardiac arrhythmias based on the FAERS database, as well as exploring the associations between concurrent cardiotoxicity and ICI-related arrhythmias, which can assist medication monitoring, clinical practice, and future investigations. Further studies are needed to address the mechanisms underlying ICI-related arrhythmias and to validate the results in our study.

## Data Availability

The original contributions presented in the study are included in the article/Supplementary Material, further inquiries can be directed to the corresponding author.
