# Supplementary material for: Cardiac arrhythmias associated with immune checkpoint inhibitors: A comprehensive disproportionality analysis of the FDA adverse event reporting system
Source: Front Pharmacol. 2022 Nov 4;13:986357. doi: 10.3389/fphar.2022.986357 (PMC9672082; doi:10.3389/fphar.2022.986357)
Supplement: Supplementary file 1 [file DataSheet1.docx]

**Table S1. Table with SMQ “Cardiac Failure” and PTs used.**

| SMQ: Cardiac Failure | | |
| --- | --- | --- |
| **PT** | Ascites | Cardiac asthma |
|  | Congestive hepatopathy | Fluid overload |
|  | Gravitational oedema | Hepatojugular reflux |
|  | Kidney congestion | Localised oedema |
|  | Oedema peripheral | Peripheral oedema neonatal |
|  | Peripheral swelling | Transfusion-related circulatory overload |
|  | Cardiac cirrhosis | Cardiac failure |
|  | Cardiac failure acute | Cardiac failure chronic |
|  | Cardiac failure congestive | Cardiac failure high output |
|  | Cardiogenic shock | Cardiohepatic syndrome |
|  | Cardiopulmonary failure | Cardiorenal syndrome |
|  | Cardio-respiratory distress | Grey syndrome neonatal |
|  | Low cardiac output syndrome | Neonatal cardiac failure |
|  | Obstructive shock | Propofol infusion syndrome |
|  | Radiation associated cardiac failure | Ventricular failure |
|  | Acute left ventricular failure | Acute pulmonary oedema |
|  | Chronic left ventricular failure | Left ventricular failure |
|  | Pulmonary congestion | Pulmonary oedema |
|  | Pulmonary oedema neonatal | Chronic right ventricular failure |
|  | Acute right ventricular failure | Cor pulmonale acute |
|  | Cor pulmonale | Kyphoscoliotic heart disease |
|  | Cor pulmonale chronic | Right ventricular failure |
|  | Pulmonary artery wall hypertrophy |  |
|  | Shoshin beriberi |  |

|  | BRASH syndrome | Cardiac fibrillation |
| --- | --- | --- |
|  | Cardiac flutter | Central bradycardia |
|  | Chronotropic incompetence | Extrasystoles |
|  | Foetal arrhythmia | Foetal heart rate acceleration abnormality |
|  | Foetal heart rate deceleration abnormality | Foetal heart rate disorder |
|  | Foetal tachyarrhythmia | Heart alternation |
|  | Holiday heart syndrome | Neonatal bradyarrhythmia |
|  | Neonatal tachyarrhythmia | Neonatal tachycardia |
|  | Nonreassuring foetal heart rate pattern | Ogden syndrome |
|  | Pacemaker generated arrhythmia | Pacemaker syndrome |
|  | Parasystole | Paroxysmal arrhythmia |
|  | Postural orthostatic tachycardia syndrome | Rebound tachycardia |
|  | Reperfusion arrhythmia | Sinusoidal foetal heart rate pattern |
|  | Tachyarrhythmia | Tachycardia |
|  | Tachycardia foetal | Tachycardia paroxysmal |
|  | Withdrawal arrhythmia | Atrial flutter |
|  | Arrhythmia supraventricular | Atrial tachycardia |
|  | Atrial fibrillation | Junctional ectopic tachycardia |
|  | Atrial parasystole | Neonatal sinus tachycardia |
|  | Congenital supraventricular tachycardia | Nodal rhythm |
|  | Neonatal sinus bradycardia | Sinus arrhythmia |
|  | Nodal arrhythmia | Sinus node dysfunction |
|  | Sinus arrest | Supraventricular extrasystoles |
|  | Sinus bradycardia | Supraventricular tachycardia |
|  | Sinus tachycardia | Cardiac arrest neonatal |
|  | Supraventricular tachyarrhythmia | Cardio-respiratory arrest |
|  | Wandering pacemaker | Pulseless electrical activity |
|  | Accelerated idioventricular rhythm | Sudden cardiac death |
|  | Cardiac arrest | Torsade de pointes |
|  | Cardiac death | Ventricular asystole |
|  | Cardio-respiratory arrest neonatal | Ventricular fibrillation |
|  | Rhythm idioventricular | Ventricular parasystole |
|  | Sudden death | Ventricular tachyarrhythmia |
|  | Ventricular arrhythmia |  |
|  | Ventricular extrasystoles |  |
|  | Ventricular flutter |  |
|  | Ventricular pre-excitation |  |
|  | Ventricular tachycardia |  |

**Table S2. Table with SMQ “Coronary artery disorders” and PTs used.**

| SMQ: Coronary artery disorders | | |
| --- | --- | --- |
| **PT** | Arteriosclerosis coronary artery | Arteritis coronary |
|  | Coronary artery aneurysm | Coronary artery compression |
|  | Coronary artery dilatation | Coronary artery disease |
|  | Coronary artery dissection | Coronary artery embolism |
|  | Coronary artery insufficiency | Coronary artery occlusion |
|  | Coronary artery perforation | Coronary artery reocclusion |
|  | Coronary artery restenosis | Coronary artery stenosis |
|  | Coronary artery thrombosis | Coronary bypass stenosis |
|  | Coronary bypass thrombosis | Coronary ostial stenosis |
|  | Coronary sinus dilatation | Coronary vascular graft occlusion |
|  | Coronary vascular graft stenosis | Diabetic coronary microangiopathy |
|  | Haemorrhage coronary artery | Angina pectoris |
|  | Acute coronary syndrome | Anginal equivalent |
|  | Acute myocardial infarction | Cardiac perfusion defect |
|  | Angina unstable | Chest pain |
|  | Arteriospasm coronary | Coronary steal syndrome |
|  | Chest discomfort | Microvascular coronary artery disease |
|  | Coronary no-reflow phenomenon | Myocardial ischaemia |
|  | Kounis syndrome | Myocardial stunning |
|  | Myocardial infarction | Periprocedural myocardial infarction |
|  | Myocardial reperfusion injury | Postinfarction angina |
|  | Papillary muscle infarction | Silent myocardial infarction |
|  | Post procedural myocardial infarction | Subendocardial ischaemia |
|  | Prinzmetal angina |  |
|  | Subclavian coronary steal syndrome |  |
|  | Wellens' syndrome |  |

**Table S3. Table with SMQ “Myocardial disorders” and PTs used.**

| SMQ: Myocardial disorders | | |
| --- | --- | --- |
| **PT** | Arrhythmogenic right ventricular dysplasia | Cardiac iron overload |
|  | Cardiac steatosis | Cardiomyopathy |
|  | Cardiomyopathy acute | Cardiomyopathy alcoholic |
|  | Cardiomyopathy neonatal | Chagas' cardiomyopathy |
|  | Congestive cardiomyopathy | Diabetic cardiomyopathy |
|  | Glycogen storage disease type II | HIV cardiomyopathy |
|  | Hypertensive cardiomyopathy | Hypertrophic cardiomyopathy |
|  | Ischaemic cardiomyopathy | Kearns-Sayre syndrome |
|  | Metabolic cardiomyopathy | Non-compaction cardiomyopathy |
|  | Non-compaction cardiomyopathy | Non-compaction cardiomyopathy |
|  | Peripartum cardiomyopathy | Restrictive cardiomyopathy |
|  | Stress cardiomyopathy | Tachycardia induced cardiomyopathy |
|  | Thyrotoxic cardiomyopathy | Toxic cardiomyopathy |
|  | Viral cardiomyopathy | Coxsackie myocarditis |
|  | Coxsackie carditis | Enterovirus myocarditis |
|  | Cytomegalovirus myocarditis | Myocardiac abscess |
|  | Malarial myocarditis | Myocarditis helminthic |
|  | Myocarditis bacterial | Myocarditis meningococcal |
|  | Myocarditis infectious | Myocarditis septic |
|  | Myocarditis mycotic | Myocarditis toxoplasmal |
|  | Myocarditis syphilitic | Atrial enlargement |
|  | Viral myocarditis | Atrial rupture |
|  | Acquired cardiac septal defect | Atrial septal defect acquired |
|  | Atrial hypertrophy | Atrioventricular septal defect |
|  | Atrial septal defect | Cardiac aneurysm |
|  | Atrio-oesophageal fistula | Cardiac pseudoaneurysm |
|  | Cardiac amyloidosis | Cardiac septal defect |
|  | Cardiac hypertrophy | Cardiac septal hypertrophy |
|  | Cardiac sarcoidosis | Cardiomegaly |
|  | Cardiac septal defect residual shunt | Diastolic dysfunction |
|  | Cardiac ventricular scarring | Dilatation ventricular |
|  | Chordae tendinae rupture | Interventricular septum rupture |
|  | Dilatation atrial | Left atrial dilatation |
|  | Holt-Oram syndrome | Left atrial hypertrophy |
|  | Ischaemic contracture of the left ventricle | Left ventricular dilatation |
|  | Left atrial enlargement | Left ventricular enlargement |
|  | Left ventricular diastolic collapse | Left ventricular hypertrophy |
|  | Left ventricular dysfunction | Myocardial calcification |
|  | Left ventricular false tendon | Myocardial fibrosis |
|  | Left-to-right cardiac shunt | Myocardial hypoxia |
|  | Myocardial depression | Myocardial oedema |
|  | Myocardial haemorrhage | Myoglobinaemia |
|  | Myocardial necrosis | Papillary muscle disorder |
|  | Myocardial rupture | Papillary muscle rupture |
|  | Myoglobinuria | Rhabdomyoma |
|  | Papillary muscle haemorrhage | Right atrial enlargement |
|  | Post cardiac arrest syndrome | Right ventricular diastolic collapse |
|  | Right atrial dilatation | Right ventricular dysfunction |
|  | Right atrial hypertrophy | Right ventricular false tendon |
|  | Right ventricular dilatation | Sigmoid-shaped ventricular septum |
|  | Right ventricular enlargement | Systemic right ventricle |
|  | Right ventricular hypertrophy | Univentricular heart |
|  | Single atrium | Ventricular compliance decreased |
|  | Systolic dysfunction | Ventricular dyskinesia |
|  | Ventricle rupture | Ventricular enlargement |
|  | Ventricular dysfunction | Ventricular hypertrophy |
|  | Ventricular dyssynchrony | Ventricular remodelling |
|  | Ventricular hyperkinesia | Ventricular septal defect acquired |
|  | Ventricular hypokinesia | Eosinophilic myocarditis |
|  | Ventricular septal defect | Hypersensitivity myocarditis |
|  | Autoimmune myocarditis | Lupus myocarditis |
|  | Giant cell myocarditis | Myocarditis post infection |
|  | Immune-mediated myocarditis |  |
|  | Myocarditis |  |
|  | Radiation myocarditis |  |

**Table S4. Table with SMQ “Pericardial disorders” and PTs used.**

| SMQ: Pericardial disorders | | |
| --- | --- | --- |
| **PT** | Atypical mycobacterium pericarditis | Bacterial pericarditis |
|  | Coxsackie pericarditis | Cytomegalovirus pericarditis |
|  | Infective pericardial effusion | Pericarditis amoebic |
|  | Pericarditis fungal | Pericarditis gonococcal |
|  | Pericarditis helminthic | Pericarditis histoplasma |
|  | Pericarditis infective | Pericarditis meningococcal |
|  | Pericarditis mycoplasmal | Pericarditis rheumatic |
|  | Pericarditis syphilitic | Pericarditis tuberculous |
|  | Purulent pericarditis | Viral pericarditis |
|  | Autoimmune pericarditis | Pericarditis |
|  | Pericarditis adhesive | Pericarditis constrictive |
|  | Pericarditis lupus | Pericarditis malignant |
|  | Pericarditis uraemic | Pleuropericarditis |
|  | Postpericardiotomy syndrome | Cardiac tamponade |
|  | Benign pericardium neoplasm | Intrapericardial thrombosis |
|  | Dressler's syndrome | Pericardial calcification |
|  | Malignant pericardial neoplasm | Pericardial effusion |
|  | Pericardial disease | Pericardial fibrosis |
|  | Pericardial effusion malignant | Pericardial lipoma |
|  | Pericardial haemorrhage | Pericardial neoplasm |
|  | Pericardial mass | Pneumopericardium |
|  | Pericardial rub |  |
|  | Radiation pericarditis |  |

**Table S5. Table with SMQ “Cardiac valve disorders” and PTs used.**

| SMQ: Cardiac valve disorders | | |
| --- | --- | --- |
| **PT** | Aortic annulus rupture | Aortic valve atresia |
|  | Aortic valve calcification | Aortic valve disease |
|  | Aortic valve disease mixed | Aortic valve incompetence |
|  | Aortic valve prolapse | Aortic valve sclerosis |
|  | Aortic valve stenosis | Aortic valve thickening |
|  | Bicuspid aortic valve | Congenital aortic valve incompetence |
|  | Congenital aortic valve stenosis | Degenerative aortic valve disease |
|  | Heyde's syndrome | Paravalvular aortic regurgitation |
|  | Subvalvular aortic stenosis | Supravalvular aortic stenosis |
|  | Unicuspid aortic valve | Williams syndrome |
|  | Carcinoid heart disease | Cardiac valve abscess |
|  | Cardiac valve discolouration | Cardiac valve disease |
|  | Cardiac valve replacement complication | Cardiac valve rupture |
|  | Cardiac valve sclerosis | Cardiac valve thickening |
|  | Cardiac valve vegetation | Congenital heart valve disorder |
|  | Congenital heart valve incompetence | Degenerative multivalvular disease |
|  | Heart valve calcification | Heart valve incompetence |
|  | Heart valve stenosis | Lambl's excrescences |
|  | Prosthetic cardiac valve thrombosis | Shone complex |
|  | Structural valve deterioration | Congenital mitral valve stenosis |
|  | Congenital mitral valve incompetence | Ischaemic mitral regurgitation |
|  | Degenerative mitral valve disease | Mitral perforation |
|  | Mitral face | Mitral valve calcification |
|  | Mitral valve atresia | Mitral valve disease mixed |
|  | Mitral valve disease | Mitral valve incompetence |
|  | Mitral valve hypoplasia | Mitral valve sclerosis |
|  | Mitral valve prolapse | Mitral valve thickening |
|  | Mitral valve stenosis | Parachute mitral valve |
|  | Myxomatous mitral valve degeneration | Congenital pulmonary valve atresia |
|  | Systolic anterior motion of mitral valve | Pulmonary valve calcification |
|  | Bicuspid pulmonary valve | Pulmonary valve incompetence |
|  | Congenital pulmonary valve disorder | Pulmonary valve stenosis |
|  | Pulmonary valve disease | Pulmonary valve thickening |
|  | Pulmonary valve sclerosis | Congenital tricuspid valve incompetence |
|  | Pulmonary valve stenosis congenital | Degenerative tricuspid valve disease |
|  | Congenital tricuspid valve atresia | Tricuspid valve calcification |
|  | Congenital tricuspid valve stenosis | Tricuspid valve incompetence |
|  | Straddling tricuspid valve | Tricuspid valve sclerosis |
|  | Tricuspid valve disease | Tricuspid valve thickening |
|  | Tricuspid valve prolapse |  |
|  | Tricuspid valve stenosis |  |
